# Nuclear sequences of mitochondrial origin in domestic yak

**DOI:** 10.1038/s41598-024-61147-7

**Published:** 2024-05-03

**Authors:** Mélissa Poncet, Maureen Féménia, Clémence Pierre, Mathieu Charles, Aurélien Capitan, Arnaud Boulling, Dominique Rocha

**Affiliations:** 1 INRAE, AgroParisTech, GABI, Université Paris-Saclay, 78350 Jouy-en-Josas, France; 2 INRAE, SIGENAE, 78350 Jouy-en-Josas, France

**Keywords:** Genomics, Mitochondrial genome

## Abstract

Mitochondrial DNA sequences are frequently transferred into the nuclear genome, generating nuclear mitochondrial DNA sequences (NUMTs). Here, we analysed, for the first time, NUMTs in the domestic yak genome. We obtained 499 alignment matches covering 340.2 kbp of the yak nuclear genome. After a merging step, we identified 167 NUMT regions with a total length of ~ 503 kbp, representing 0.02% of the nuclear genome. We discovered copies of all mitochondrial regions and found that most NUMT regions are intergenic or intronic and mostly untranscribed. 98 different NUMT regions from domestic yak showed high homology with cow and/or wild yak genomes, suggesting selection or hybridization between domestic/wild yak and cow. To rule out the possibility that the identified NUMTs could be artifacts of the domestic yak genome assembly, we validated experimentally five NUMT regions by PCR amplification. As NUMT regions show high similarity to the mitochondrial genome can potentially pose a risk to domestic yak DNA mitochondrial studies, special care is therefore needed to select primers for PCR amplification of mitochondrial DNA sequences.

## Introduction

The evolution and function of eukaryotic genomes have strongly been influenced by the integration of mitochondrial DNA sequences into the nuclear genome^1^. Because of their homology, these nuclear mitochondrial DNA sequences, known as NUMTs, can compromise mitochondrial DNA studies if they are not taken into account. Indeed, the inadvertent analysis of NUMTs as mitogenomic sequences can lead to misleading results in the diagnosis of mitochondrial diseases, phylogenetic reconstructions, population studies and DNA barcoding analyses^1,2^. For example, Yao and collaborators revisited mitochondrial DNA sequence variations which were caused by accidental amplification of NUMTs^2^. They described several cases, included one NUMT sharing homologies with mitochondrial *ND5* gene and generating a false mitochondrial variant believed to be responsible for hearing loss. It is therefore important to identify nuclear sequences of mitochondrial origin.

To date, a catalogue of NUMTs has been established for a large number of different avian and mammalian species^3–9^ including several ruminant species^10–12^, but not yet in the domestic yak (*Bos grunniens*). For example, we identified 166 bovine and 390 ovine NUMT regions, representing approximately 0.02% of the nuclear genome in each species^10,11^.

The domestic yak is a very important species for the inhabitants of the Qinghai-Tibetan Plateau and the Himalayan main range, providing food (milk and meat), fibre, fertiliser and carrying heavy loads. As a result, a reference genome sequence of the domestic yak has been available since 2012^13^ and several studies have been conducted to describe the genetic diversity of domestic yak populations based on mitochondrial DNA sequences^14–16^.

Based on the analysis of whole-genome sequences of 59 domestic and 13 wild yaks, Qiu and colleagues estimated that domestication began about 7300 years ago^17^. Genetic introgression between domestic yaks and cattle has been reported^18–23^. NUMTs in domestic yak can possibly affect the previous domestication and introgression studies.

In this paper, we present the first study of NUMTs in the domestic yak genome. This study will therefore allow a more accurate analysis of the mitogenome of this yak species.

## Material and methods

### Data sources

The mitochondrial reference sequence and the latest BosGru3.0 domestic yak reference genome sequence^24^ were obtained from Genbank (assembly accessions: NC_006380.3 and GCA_005887515.1, 10th June 2019 respectively). Reference genome sequences of wild yak (*Bos mutus*) BosGru_v2.0^17^ and cow genome (*Bos taurus*) ARS-UCD1.3 (Genbank assembly accessions: GCA_000298355.1, 9th January 2013 and GCA_002263795.4, 1st July 2023, respectively) were also retrieved from Genbank.

### Genome-wide identification of NUMT regions

To deal with the fact that the mitochondrial genome is circular, we perform alignments on the nuclear genome of the domestic yak using two different linearised sequences of the mitogenome: (1) a standard linearisation starting at position 1 and ending at position 16,323; and (2) a shifted linearisation starting arbitrarily at position 5001 and ending at position 5000. This allowed the identification of nuclear genome sequences that overlapped both ends of the standard linear mitochondrial genome sequence. These two linearised mitogenome sequences were aligned to the domestic yak genome sequence using BLASTN^25^, following the procedure described by Calabrese et al.^3^. However, an e-value threshold of 10^–4^, comparable to other studies of NUMTs^1^, was applied. NUMTs that were not more than 10 kbp apart on the nuclear genome were merged into one NUMT region^10,11,26^.

### Characterization of NUMT regions

Gene annotation of the domestic yak genome was retrieved from ENSEMBL (release 106). To explore if identified NUMT regions are transcribed, we performed blastn alignments with *Bos grunniens* sequences from GenBank refseq_rna and EST databases. NUMTs detected on unplaced scaffolds have been excluded. In order to better investigate the possible transcription, we use ORF Finder^27^ to predict open reading frames (ORFs) in the sequence of NUMT regions that have been identified. We further annotated the predicted full-length ORFs using the CD search tool^28^.

### Experimental validation of NUMT regions

PCR primers were designed within the flanking regions of the NUMT regions using primer-blast ^29^. In addition, each primer sequence was aligned using blastn onto the domestic yak reference genome sequence and the mitochondrial genome sequence in order to verify its nuclear specificity. Primers were purchased from Integrated DNA Technologies and sequences can be found in Supplementary material—Table S1. Polymerase chain reactions were done as previously described^10^. Briefly, PCRs were performed in 10 μl, using 50 ng of genomic DNA from a male domestic yak of Mongolian origins, 1 U Go*Taq* DNA polymerase (Promega), 1X PCR buffer, 1.5 mM MgCl_2_, 200 μM of each dNTP and 1.0 μM of each PCR primer. The following touchdown cycling protocol was used: 95 °C for 2 min followed by 13 cycles of 95 °C for 1 min, 1 min of annealing (the annealing temperature was progressively lowered from 68 to 56 °C in steps of 1 °C every cycle) and 72 °C for 2 min. These initial cycles were followed by 30 cycles of 95 °C for 1 min, 55 °C for 1 min and 72 °C for 2 min, and a final extension step at 72 °C for 5 min. PCR products were analysed by electrophoresis on an 1% agarose gel to verify the expected length of amplicons. The nucleotide sequences of the amplicons were subsequently determined using Sanger sequencing (Eurofins Genomics). All sequences were visually inspected using Chromas (Technelysium) and then aligned to the BosGru3.0 domestic yak reference genome sequence using blastn.

## Results and discussion

### NUMT catalogue in domestic yak

**Figure 1:**
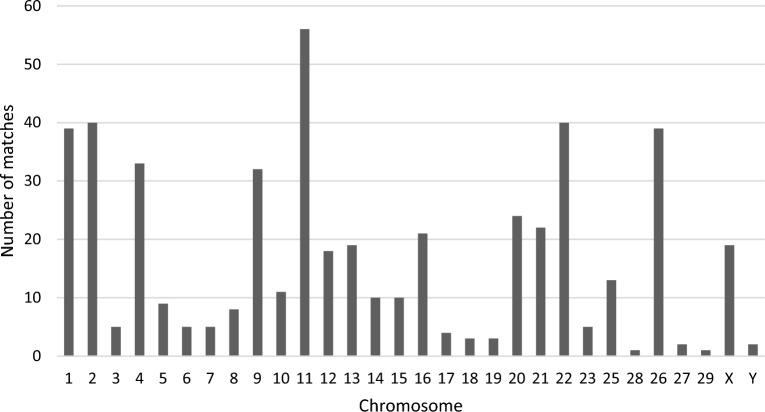
Distribution of NUMTs (alignment matches) across all chromosomes.

**Figure 2:**
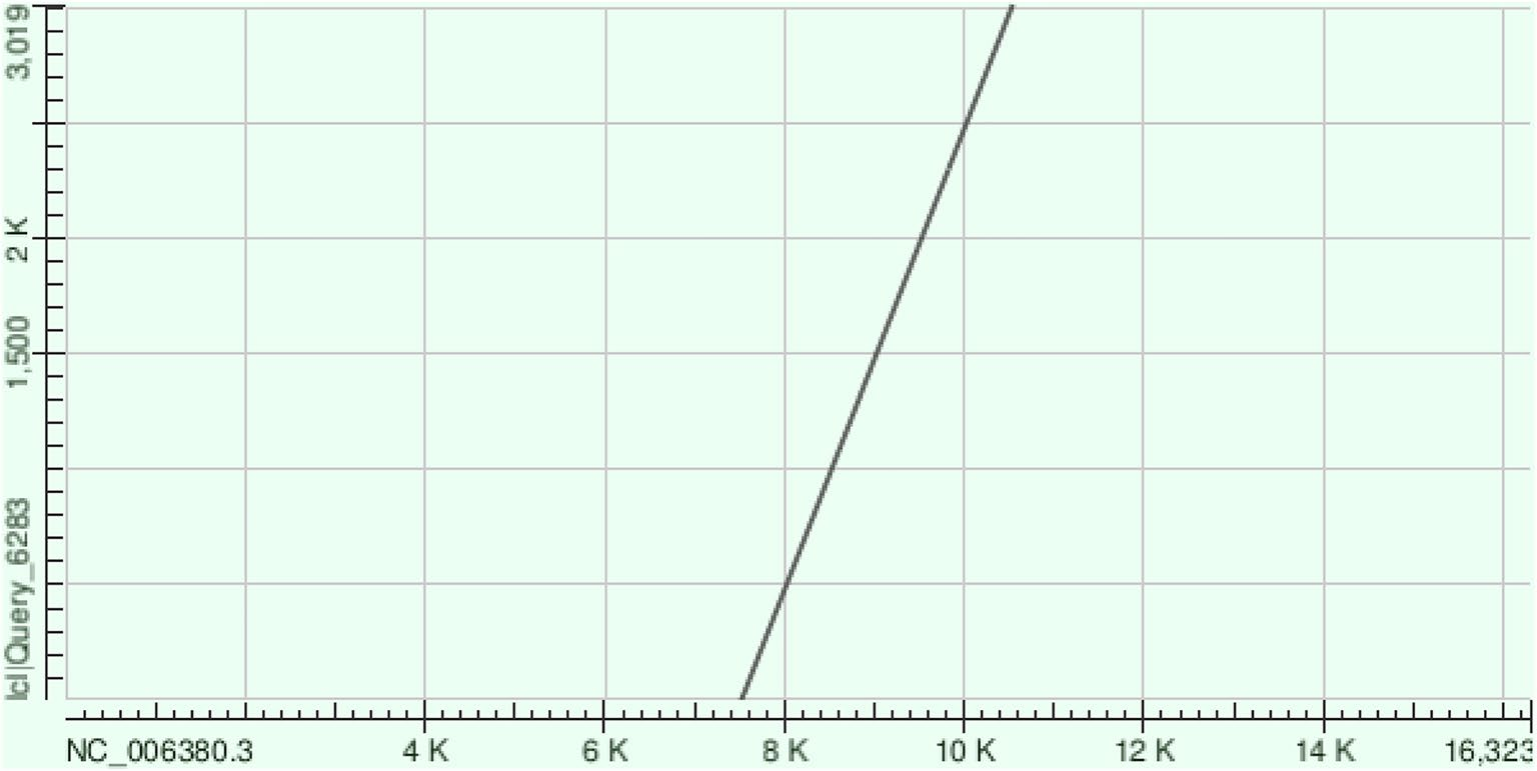
Dot plot representing the alignment of a large nuclear mitochondrial DNA sequence (Y.3.84) with the domestic yak mitogenome. Sequences of the mitochondrial genome and of the sequence of the NUMT region are plotted on *X* axis and *Y* axis respectively. The positions indicated in the axes of the dot plot start at 1 and go to the complete length of the sequence. Therefore, dot plot representation is not on the same scale for the *X* and *Y* axes.

**Figure 3:**
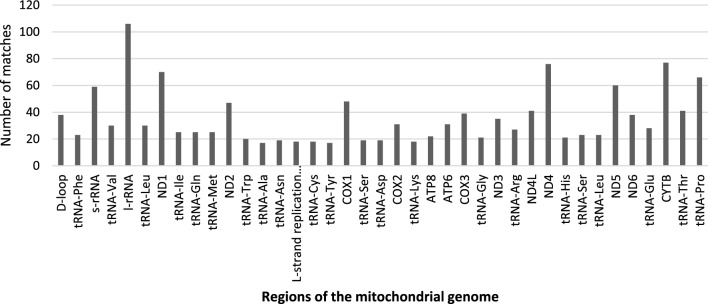
Number of nuclear mitochondrial DNA sequences containing, completely or partially, each region of the domestic yak mitogenome.

Alignment matches between the domestic yak genome sequence and the two linearised mitogenome sequences were detected in all chromosomes, including the Y chromosome (Fig. 1). We identified 499 NUMTs (alignment matches), with similarity between nuclear and mitochondrial sequences ranging between 62.9 and 100%. The total length of the alignment was 340.2 kbp, with alignment matches ranging from 31 to 11,900 bp. About 13% of the alignment matches between mitochondrial and nuclear sequences show sequence similarity higher than 85%. For example, a contiguous alignment match of 3,018 bp with similarity of 89.6% was found between region 98,761,848–98,764,866 of chromosome 3 and the mitogenome (position 7520–10,540) (Fig. 2). Mitochondrial regions present in each NUMT were defined based on the positions of the alignment matches in the mitogenome. All mitochondrial genes had alignment matches with NUMTs, however distinct numbers of copies were detected (Fig. 3). Some of the most occurring fragments of the mitogenome in the nuclear genome included the *D*-loop control region and neighbouring genes (i.e. *l-rRNA*, *ND1*, *CytB*). While the mechanisms of mitochondrial DNA integration into the nuclear genome are not yet fully understood, interestingly, Rothfuss et al.^30^ have shown in a human neuroblastoma cell line that the *D*-loop region is more prone to breakage than the rest of the mitochondrial genome. Moreover, Doynova et al*.*^31^ have showed that the *D*-loop region has a greater propensity for interaction with the nuclear genome, in cultured human and mouse cells. All these results suggest that the high number of nuclear sequences originating from the mitochondrial *D*-loop region may be arise from a higher number of direct integrations into the nuclear genome.

Nuclear copies of mitochondrial origin can be highly modified after integration by insertions and deletions. Consequently, NUMTs that were no more than 10 kbp apart on the nuclear genome were merged into one NUMT region. After this merging step, we identified a total of 167 NUMT regions ranging from 31 to 35,446 bp (Supplementary material—Table S2 and Fig. S1). We identified 35 NUMT regions with more than 5000 bp, representing ~ 21% of the total number of NUMTs regions and around 80% of the NUMT region total length. By contrast, NUMT regions smaller than 300 bp comprise ~ 45% of the total NUMT regions length, but represent only ~ 2% of the NUMT region total length. The total length of the NUMT regions reached 502.6 kbp, representing 0.02% of the domestic yak nuclear genome. This represents almost a twofold increase of the total length detected after merging co-linear NUMTs. Interestingly we also found the same proportion of 0.02% of the genome including NUMT regions in cattle^10^ and sheep^11^.

### NUMT conservation among cattle, domestic and wild yaks

To investigate the conservation of the NUMT regions identified in the domestic yak, their sequences were aligned, after masking repeats, to the wild yak (*Bos mutus*) and cow genomes. 36,964 and 206,374 hits (e-value threshold of 10^–4^) were obtained with the wild yak and cow genomes, respectively. Only the 35 bp long NUMT Y.17.344 did not match any species. 82 different NUMT regions from the domestic yak showed high homology (identity percentage > 90%) with the wild yak and cow genomes. 16 NUMT regions were shared only by both yak species. Interestingly, 32 NUMT regions were highly conserved (> 99% identity) among the three species (Supplementary Material—Table S3). This high conservation could be due to selection or hybridisation between domestic/wild yak and cow. Indeed, hybridisation between domestic and wild yaks or between cattle and domestic yaks has been reported^32^. Domestic yak and cattle (*Bos taurus*) are capable of producing offspring. F1 hybrid males are sterile, but F1 hybrid females are fertile and can be backcrossed to cattle or yak bulls^18^. Several studies have described the introgression of bovine sequences into domestic yak genomes^18–23^.

### Characterization and in silico analysis of the expression of nuclear copies of mitochondrial genes

We compared the location of the 167 NUMT regions using the latest gene annotation of the domestic yak. NUMTs are mostly located in non-genic regions. We identified only 48 NUMT regions overlapping with 46 genes (45 NUMT regions) and 3 pseudogenes (3 NUMT regions; Supplementary material—Table S4). We found that 31 NUMT regions overlapping with genes are located in introns. For example, NUMT region Y1.7 is located within intron 3 of *TNFSF10*. All remaining NUMTs contains 5′ or 3′ untranslated regions, including sometime part of the first exon. In addition, one NUMT (Y.10.193), located on chromosome 10 and 10.7 kb long encompasses the full sequence of a novel single-exon gene (*ENSBGRG00000022824*) encoding a 112 amino-acid protein. This gene is conserved in several mammalian species, including American bison and zebu.

To investigate if these mitochondrial nuclear sequences are transcribed, we performed blastn alignments with refseq_rna and EST databases from GenBank of *Bos grunniens*. NUMTs found on unplaced scaffolds were excluded. We identified significant blast hits (*e* value threshold of 10^−4^ and identity of at least 98%) with refseq_rna sequences for 12 NUMT regions. These sequences match 12 predicted non-coding RNA sequences, although some of these sequences are transcript isoforms of the same gene. (Supplementary material—Table S5). Interestingly none of these refseq_rna sequences share significant homology to the mitochondrial genome, however at least six of these refseq_rna sequence contain domains associated to mitochondrial proteins (e.g. cytochrome P450 domain). We did not find significant matches between the sequences of NUMTs and ESTs. However, only 74 ESTs from domestic yak are currently deposited in NCBI databases, all generated from a single mammary gland sample. The results of these sequence comparisons suggest the possibility that some of the NUMT regions we identified contain elements that might be transcribed.

In order to further examine the possible transcription of the identified NUMTs, we predicted with ORF Finder open reading frames located within the sequences of each NUMT region. 7220 ORFs were predicted, ranging from 29 to 1286 amino acids. We found 258 partial ORFs, containing a start codon but without a stop codon, among these ORFs. The remaining part of those predicted ORFs may be found in the nuclear sequences flanking these NUMT regions. We found 110 different conserved protein domains, within 175 ORFs from 61 NUMT regions. As expected, most of these protein domains are shared with proteins encoded by the mitochondrial genome (e.g. CYTB domain).

### Experimental validation of NUMT regions

**Figure 4:**
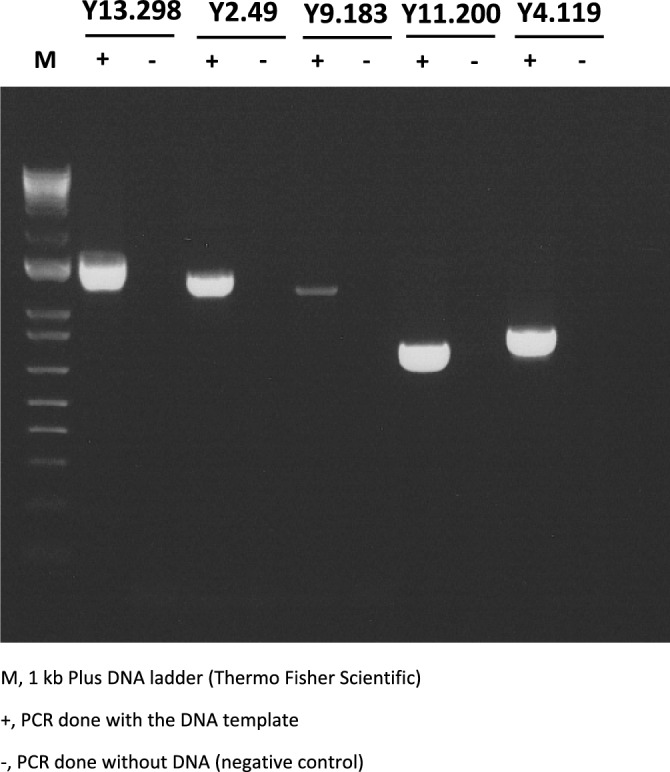
PCR amplification of 5 NUMT regions with genomic DNA.

In order to exclude the possibility that the identified NUMTs could be artefacts of the latest domestic yak genome assembly, we randomly selected five NUMT regions located on five different chromosomes for experimental validation. All five amplicons were of the expected length supporting the absence of genome assembly artefacts (Fig. 4). The nucleotide sequence of each amplicon was subsequently partially determined using Sanger sequencing (Eurofins Genomics). blastn alignment of the amplicon sequences to the domestic yak reference genome sequence confirmed the presence of these NUMT regions.

## Conclusion

Our study provides the first comprehensive description of NUMTs in the domestic yak genome. Interestingly we found 34 NUMTs highly conserved among domestic yak, wild yak and cattle, suggesting hybridization between these species or that these regions are under selection. We found several NUMT regions showing high similarity to the mitochondrial genome that could potentially pose a risk to mitochondrial studies. For example, we discovered that a portion of NUMT region Y.26.469 located on chromosome 26 shares > 97% identity with a mitochondrial sequence containing the *D*-loop control region, which is often used in genetic diversity and phylogenetic studies^33^.

Therefore special care should be taken when selecting primers for PCR amplification of mitochondrial DNA.

### Supplementary Information

Supplementary Information.

Supplementary Table S2.

Supplementary Table S3.

Supplementary Table S4.

Supplementary Table S5.

## Data Availability

Analyses used as raw data publicly available data (i.e. the genome reference sequences of the domestic yak, of the wild yak and of the cow, in addition to the domestic yak mitochondrial sequence, see “Material and method” section). All data supporting the findings of this study are available as part of the article and no additional source data are required.
