# Supplementary material for: Nuclear sequences of mitochondrial origin in domestic yak
Source: Sci Rep. 2024 May 3;14:10217. doi: 10.1038/s41598-024-61147-7 (PMC11068780; doi:10.1038/s41598-024-61147-7)
Supplement: Supplementary file 1 — Supplementary Information. [file 41598_2024_61147_MOESM1_ESM.pdf]

Supplementary information

Figure S1

Y.1.34 (multiple insertions)

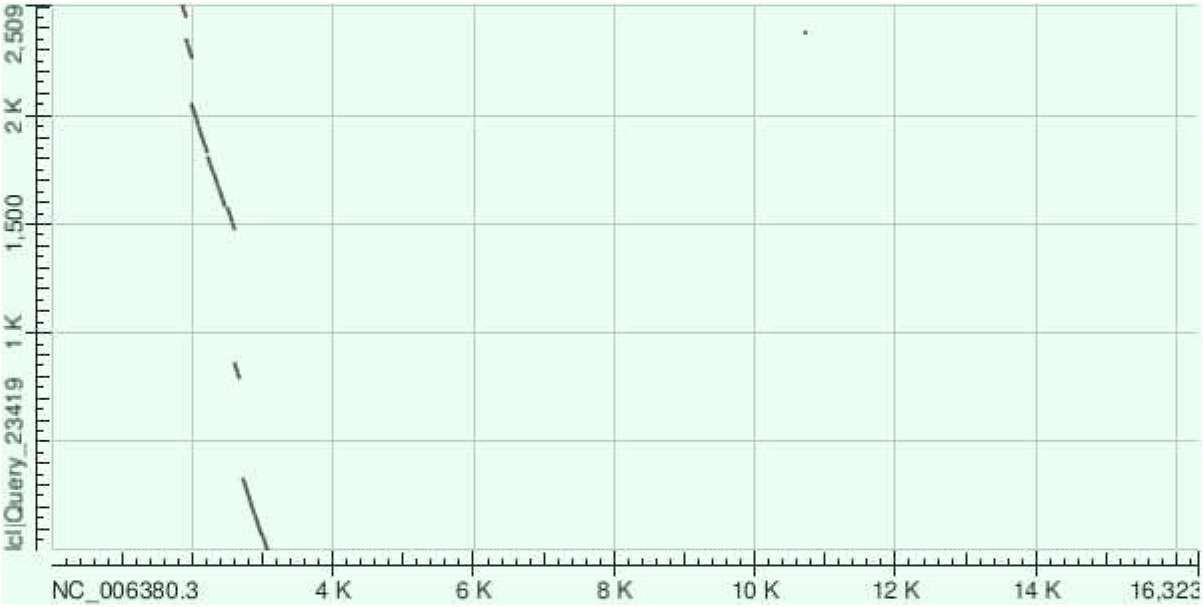

Y.2.73 (multiple insertions and duplications)

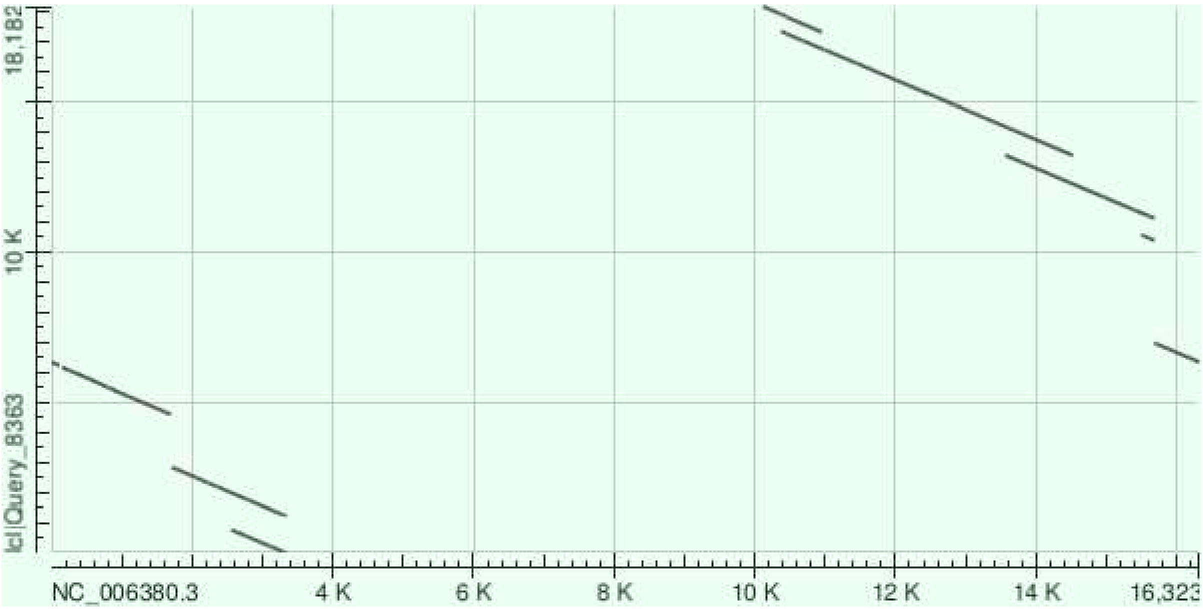

Fig. S1. Dot plots of highly modified NUMT regions (upper, NUMT Y.1.24 and bottom Y.2.73). Sequences of the mitochondrial genome and of the NUMT regions are plotted on X axis and Y axis, respectively. The positions indicated in the axes of the dot plot start at 1 and go to the complete length of the sequence. Therefore, dot plot representations are not on the same scale for the X and Y axes.

Please note that the alignment of NUMT Y.2.73 is split into two large fragments due to the circular configuration of the mitochondrial genome.

NUMT	Sequence forward primer	Tm (°C)	Sequence reverse primer	Tm (°C)	Amplicon size (bp)
Y13.298	TGTGGCTGGCGAGTCCCTC	61	ACCCCTGTTTCTCAAGGCCTCT	60	1623
Y2.49	TAGAAATTCCTTAACTTGTTGAGG	51	TTCAAAGAGTAATGCATCCTAACCC	55	1340
Y9.183	ATGGGGAAACAAATAGGAAGAT	52	ACTGATGATGCTAGTGGTCCT	55	1183
Y11.200	ACCCCGCCCTGTATGTGCTC	61	GGAAATCTGATGTTATGCAAAGCCA	57	757
Y4.119	TGTTGGAGGAGGGAACGGGC	61	GCATGACCCAGGGCTGACTT	59	850

Table S1. Information on primers used for the PCR validation.

Table S2. List of NUMT regions detected in the domestic yak genome assembly.

Table S3. Highly conserved NUMT regions detected in domestic yak, wild yak and cow.

Sequences with 100% identity are highlighted in green, whereas the orange colour indicates duplicated sequences.

Table S4. NUMT regions located within gene boundaries.

Table S5. Summary of the BLASTN sequence comparison between NUMT's and rna-refseq sequences.
